# Barriers to implementation of pediatric emergency department interventions for parental tobacco use and dependence: a qualitative study using the theoretical domains framework

**DOI:** 10.1186/s43058-021-00251-5

**Published:** 2022-01-12

**Authors:** Ashley L. Merianos, Kayleigh A. Fiser, E. Melinda Mahabee-Gittens, Michael S. Lyons, Judith S. Gordon

**Affiliations:** 1 School of Human Services, University of Cincinnati, P.O. Box 210068, Cincinnati, OH 45221-0068 USA; 2 Center for Addiction Research, College of Medicine, University of Cincinnati, Cincinnati, OH USA; 3 Division of Emergency Medicine, College of Medicine, Cincinnati Children’s Hospital Medical Center, University of Cincinnati, 3333 Burnet Avenue, Cincinnati, OH 45229 USA; 4 Department of Emergency Medicine, College of Medicine, University of Cincinnati, 231 Albert Sabin Way, Cincinnati, OH 45267-0769 USA; 5 College of Nursing, The University of Arizona, 1305 N Martin Avenue, PO Box 210203, Tucson, AZ 85721-0203 USA

**Keywords:** Tobacco smoke exposure, Tobacco counseling, Children, Parents, Pediatric emergency department, Urgent care, Theoretical domains framework, Knowledge, Beliefs about capabilities, Environmental context and resources

## Abstract

**Background:**

Pediatric emergency department (PED) and urgent care (UC) professionals can play a key role in delivering evidence-based guidelines to address parental tobacco use and child tobacco smoke exposure (TSE). Understanding PED/UC professionals’ perceptions regarding these guidelines is the first step in developing and implementing a TSE screening and counseling intervention in these settings. This study aimed to use the theoretical domains framework (TDF) to identify current screening and counseling behaviors of PED/UC professionals related to parental tobacco use and child TSE, and determine barriers and enablers that influence these behaviors.

**Methods:**

Semi-structured, focused interviews were conducted with 29 actively practicing PED/UC clinical staff who worked at one large, Midwestern children’s hospital. The interview guide was informed by the TDF and included open-ended questions. Content analysis of interview transcripts was guided by the TDF. Nurses, physicians, and healthcare administrators were assessed overall and by group membership to ensure each group was represented based on their varying PED/UC roles.

**Results:**

Fifty-one percent were nurses, 38% were physicians, and 11% were healthcare administrators. Most PED/UC professionals did not currently follow the guidelines, but perceived addressing parental tobacco use as part of their role. All 14 TDF domains were identified by nurses, physicians, and administrators in relation to counseling for parental tobacco use and child TSE. Domains with the most sub-themes were (1) *knowledge*: lack of knowledge about tobacco counseling, including implementing counseling, cessation resources/referrals, and thirdhand smoke; (2) *beliefs about capabilities*: not comfortable counseling parents, easier to discuss with parents who are receptive and to ask and advise when patients have a TSE-related complaint, and more likely to discuss if there were resources/referrals; and (3) *environmental context and resources*: barriers include lack of time, training, and resources and referral information to give to parents, and an enabler is using TSE-related complaints as a context to offer counseling.

**Conclusions:**

Study findings provide a strong foundation for developing and implementing clinical practice guidelines regarding parental tobacco use and child TSE in the PED/UC setting. Future intervention development will address all TDF domains and test the implementation of the intervention in the PED/UC setting.

Contributions to the literature

- Literature shows there is suboptimal routine delivery of evidence-based guidelines for tobacco use and tobacco smoke exposure (TSE) screening and counseling in acute healthcare. Pediatric emergency department (PED) and urgent care (UC) patients have disproportionately high rates of TSE.
- The theoretical domains framework (TDF) was used to understand current behavior of tobacco screening and counseling among PED/UC professionals.
- This analysis identifies common barriers and enablers that may influence future intervention development and implementation in the PED/UC setting, and provides researchers with a theoretical rationale for addressing these barriers.

## Background

Tobacco use disorders impose an inordinately large public health burden on emergency departments (EDs) [1]. Parents who bring their children to the pediatric ED (PED) have high cigarette smoking rates ranging from 28% up to 48% [2, 3] compared with the US general adult population (14%) [4]. This discrepancy may be due to PEDs commonly caring for those of lower socioeconomic status (e.g., public insurance) [5], which is inversely related to adult smoking [4] and child tobacco smoke exposure (TSE) [6]. PED patients have high rates of TSE, which is defined as exposure to secondhand smoke and thirdhand smoke (i.e., aged secondhand smoke) [7]. Secondhand smoke is inhaled by children from mainstream smoke exhaled by smokers and sidestream smoke from lit tobacco products. Thirdhand smoke is inhaled, orally ingested, and/or dermally transferred from the residual tobacco smoke toxicants that are left behind in the environment after tobacco smoking has been ceased.

The PED is an important venue for child TSE reduction and other modifiable health behavior efforts since this setting is frequently used as both primary and acute care sites by vulnerable patients who do not have access to regular, outpatient primary care [8–11]. Thus, this population needs improved screening and services designed to treat parental tobacco use disorders and reduce child TSE. ED and urgent care (UC) visits constitute a “teachable moment” to promote tobacco-related behavior change [3, 12]. The emergency setting is generally underutilized for prevention interventions due to perceived lack of time and resources [13]. However, emergency care settings across the US care for a large annual volume of patients [14] and may be ideal venues for preventive care given the long patient wait times and the feasibility of implementing interventions without disrupting clinical flow [3, 15]. Although evidence-based interventions have been effective in the outpatient pediatric setting [16], more research is needed on the barriers to and enablers of effective TSE interventions in the unique PED/UC setting.

The US Preventive Services Task Force [17] strongly recommends that healthcare professionals screen all patients for tobacco use and provide brief behavioral interventions to help adult tobacco users quit smoking. The US Public Health Service’s evidence-based Clinical Practice Guideline, *Treating Tobacco Use and Dependence* [17], describes five major steps (i.e., the “5A’s”) to provide brief intervention. These steps are to (1) “ask” about tobacco, (2) “advise” users to quit, (3) “assess” willingness to make a quit attempt, (4) “assist” willing users to make a quit attempt, and (5) “arrange” to help prevent relapse via follow-up. The “5 A’s” framework is the gold standard for brief tobacco screening and intervention delivery in healthcare settings [18]. National guidelines promote the use of the evidence-based “5 A’s” to assist healthcare professionals in the assessment and delivery of treatment for tobacco use and dependence in the general healthcare [17, 19] and ED [1] settings.

PED professionals’ screening for parental tobacco use and child TSE and counseling families can reduce TSE-related illness. The US Surgeon General’s [18] recent report on smoking cessation indicates that there is sufficient evidence to conclude that the development and dissemination of evidence-based clinical practice guidelines including the “5 A’s” can increase screening for tobacco use and delivery of interventions for smoking cessation in clinical settings. Two meta-analyses of ED-initiated randomized controlled trials (RCTs) concluded that tobacco control efforts promote tobacco abstinence for up to one year [20, 21]. In addition, parents are satisfied with receipt of the “5 A’s” from PED/UC professionals during their children’s visits [15]. However, research indicates that PED/UC professionals do not regularly screen for or systematically follow evidence-based guidelines to address parental tobacco use and child TSE [3, 22–27]. There is a need to understand why there is suboptimal delivery of evidence-based guidelines by PED professionals in order to develop strategies for implementing and sustaining consistent adherence to these guidelines.

The theoretical domains framework (TDF) was designed by an expert consensus for implementation research that combines multiple behavior change theories to guide the study, development, and implementation of evidence-based guidelines [28, 29]. The TDF provides an empirical method for assessment of implementation problems and informs implementation of evidence-based practices (e.g., the “5 A’s”) in clinical settings [28]. The TDF has been used in quantitative tobacco research to assess barriers to and enablers of implementing tobacco prevention and cessation counseling guidelines [30]. A qualitative approach is most frequently used when applying the TDF to identify key behaviors important for implementation of a specific intervention and for intervention development [31]. Therefore, the TDF has also been applied to qualitative tobacco research to gain a better understanding of clinical behaviors related to providing smoking cessation support [32, 33]. This framework was used in the present qualitative study to identify aspects of healthcare professionals’ behavior as the first step in adapting and implementing an evidence-based “5 A’s” intervention in the PED/UC setting.

The present study aimed to identify current screening and counseling behaviors of PED/UC nurses, physicians, and healthcare administrators related to parental tobacco use and child TSE and determine barriers and enablers that influence current behavior of delivering evidence-based tobacco counseling. Evidence-based guidelines suggest a systematic approach to developing and implementing TSE interventions [18], and most “5 A’s” interventions are delivered using a team-based approach, which involves all members of the healthcare team. Therefore, all three professional groups (nurses, physicians, and administrators) were assessed to ensure all potential team members were represented based on their varying professional roles in the PED/UC setting.

## Methods

### Study design and setting

The study used semi-structured, focused qualitative interviews with PED/UC professionals who work at one large, Midwestern tertiary care children’s hospital. There are two PEDs and five UCs associated with the hospital that have a collective annual volume of over 150,000 patients, making it one of the busiest in the US. All confidential interviews were conducted and recorded virtually using a secure, research compliant, Internet-based conferencing tool provided by the principal investigator’s institution. This study used the Standards for Reporting Qualitative Research (SRQR) items to follow reporting guidelines for qualitative research. Ethical approval for this study was obtained from the University of Cincinnati (institutional review board [IRB] number: 2020-0207) and Cincinnati Children’s Hospital Medical Center (IRB number: 2020-0248).

### Participants

Participants were 29 clinical staff with direct patient contact who worked in the PED/UC at the children’s hospital. A stratified purposive sample was recruited to ensure views of all professional groups were represented in this study [34]. Participants were limited to the first 30 interested and eligible clinical staff. This included 16 nurses (registered nurses and nurse practitioners), 10 physicians (medical doctors and doctors of osteopathic medicine), and four healthcare administrators (clinical managers and directors). One nurse withdrew, and therefore, a total of 15 nurses were interviewed. A recruitment email was sent to a total of 297 nurses and 76 physicians by the principal investigator via three hospital email listservs to personally invite PED/UC professionals from all areas of clinical practice to participate in the study. Professionals who were interested in participating were instructed to email the principal investigator for more information. The investigator emailed interested and eligible participants who responded with a research information sheet that outlined study details and potential scheduling times for the one-hour virtual interview. Following standard focused interview recommendations [34], PED/UC professionals who consented to participate were individually interviewed until “saturation” (i.e., where no new information emerged) was reached among all professional groups.

### Procedure

All interviews were conducted virtually due to COVID-19 restrictions. The principal investigator attended all 29 interviews, introduced the interview study purpose, asked eligible participants if they had any questions about the research information sheet they received via email before participation, and reminded them that they could stop participation at any time. All participants provided verbal consent to participate and to be recorded. Participants received $50 compensation for their time and effort in the form of a reloadable debit card that was mailed to their homes.

From April 28, 2020 to May 5, 2020, the principal investigator conducted 11 interviews alone, and 18 interviews with another trained study team member (KAF). While the study team member led the 18 interviews, the principal investigator was able to take notes, answer study questions, and ask clarifying questions. Upon interview completion, the principal investigator ordered mechanical transcriptions of the virtual recordings, which were about 70–80% accurate. Then the study team member used the mechanical transcriptions as a starting point to transcribe each interview verbatim, and finally, removed any potentially identifiable information.

### Interview topic guide

A semi-structured interview guide was developed by the research team, which had expertise in qualitative methods, behavior change, clinical and translational research, emergency medicine, and implementation science. The team consisted of two professors with doctoral degrees in either health education or clinical psychology, two practicing ED and PED/UC medical doctors, and one doctoral-level research assistant. The guide was informed by the TDF, which has 14 theoretical domains derived from 33 validated theories [28, 29]. The overarching aim of the TDF is to identify elements essential for implementation outcomes [28, 29], and is highly correlated with the development and implementation of quality, clinical interventions [35]. The interview guide was piloted during the first two interviews and revised by the principal investigator. Table 1 presents the TDF domains defined by Cane et al. [28], and corresponding interview questions.

**Table 1:** Semi-structured interview guide questions and corresponding TDF domains

TDF domain	TDF domain definition^**a**^	Interview questions
Knowledge	An awareness of the existence of something	Do you know about the Clinical Practice Guideline for Treating Tobacco Use and Dependence (the “5 A’s”)?
Skills	An ability or proficiency acquired through practice	How do you currently identify parental or household smokers? What do you do to help them take an active role in reducing their child’s exposure to tobacco smoke?
Social/professional role and identity	A coherent set of behaviors and displayed personal qualities of an individual in a social or work setting	What aspects of reducing patients’ secondhand smoke exposure do you see as part of your role? Which types of healthcare professionals do you think should be involved in reducing patients’ secondhand smoke exposure?
Beliefs about capabilities	Acceptance of the truth, reality, or validity about an ability, talent, or facility that a person can put to constructive use	What special skills or expertise, if any, would you need to reduce patients’ secondhand smoke exposure? What makes it easy to counsel parents or household smokers on reducing patients’ secondhand smoke exposure?
Optimism	The confidence that things will happen for the best or that desired goals will be attained	Currently, how effective are healthcare providers at screening and counseling secondhand smoke-exposed patients and their families?
Beliefs about consequences	Acceptance of the truth, reality, or validity about outcomes of a behavior in a given situation	What do you think might happen clinically to the patient if healthcare providers do not take steps to screen or counsel patients who are exposed to secondhand smoke?
Reinforcement	Increasing the probability of a response by arranging a dependent relationship, or contingency, between the response and a given stimulus	Is there anything that you think would encourage or discourage healthcare providers from screening or counseling parental or household smokers?
Intentions	A conscious decision to perform a behavior or a resolve to act in a certain way	What would help to make screening and counseling a priority to healthcare providers?
Goals	Mental representations of outcomes or end states that an individual wants to achieve	Is screening and counseling a routine part of your job or is it something you need to take time to think about? Do you screen when you “smell smoke” in the room or if a patient has a specific illness like asthma?
Memory, attention, and decision processes	The ability to retain information, focus selectively on aspects of the environment, and choose between two or more alternatives	What thought processes might guide your decision to screen for secondhand smoke exposure and provide counseling to patients and their families?
Environmental context and resources	Any circumstance of a person’s situation or environment that discourages or encourages the development of skills and abilities, independence, social competence, and adaptive behavior	What factors in your working environment do you think influence whether you are able to screen for secondhand smoke exposure and provide counseling to parents and household members?
Social influences	Those interpersonal processes that can cause individuals to change their thoughts, feelings, or behaviors	If you want to increase the frequency in which healthcare providers screen for secondhand smoke exposure and reduce patient exposure through counseling, how would you do this?
Emotion	A complex reaction pattern, involving experiential, behavioral, and physiological elements, by which the individual attempts to deal with a personally significant matter or event	No specific interview question.
Behavioral regulation	Anything aimed at managing or changing objectively observed or measured actions	Are certain incentives or other things needed for screening and counseling patients and their families?

^a^Definitions taken directly from Cane et al. [28]

### Data analysis

Directed content analysis of qualitative data was guided by the TDF, and data were categorized into the individual TDF domains [31]. The principal investigator (ALM) and a trained study team member (KAF) who co-conducted the interviews started with five transcripts that were randomly selected. The two researchers independently read each transcript, open coded the transcript text, and generated sub-themes that were allocated to the 14 TDF domains. If > 2 TDF domains were relevant while coding, then they were initially cross-indexed to both domains. After completion of the first five transcripts, the researchers met to discuss their coding and resolve any disagreements and reached consensus on which domain should be selected to best reflect any cross-indexed text, based on the best match to the TDF definition for each domain (see Table 1). If consensus could not be reached, a third study team member (JSG) was available to resolve the conflict. An audit trail was used to define codes and document coding decisions including each TDF domain, sub-theme, and related quotes. It was noted whether sub-themes arose from participants overall, and by professional group. The remaining 24 interviews were independently coded in a random fashion by the same two investigators using the coding guideline. Once the coding process was complete, all interview transcripts were reviewed a second time to enhance analytical rigor and ensure important sub-themes and quotes were not originally overlooked or misapplied.

## Results

Participant characteristics overall, and by professional group, are summarized in Table 2. On average, participants worked 35 hours per week and cared for 51 patients per week. Participants had worked an average of eight years in their current position, and over 14 years at the hospital in any position. Only one participant had received training on tobacco counseling in the past year.

**Table 2:** PED/UC professional characteristics overall and by professional group

Characteristic	Overall (***N***=29) ***n***^**a**^	Nurse (***n***=15) ***n***^**a**^	Physician (***n***=10) ***n***^**a**^	Administrator (***n***=4) ***n***^**a**^
**Age,** ***M*** **(SD)**	42.4 (10.1)	38.5 (10.0)	44.0 (6.6)	52.8 (11.3)
**Sex**				
Male	5	0	3	2
Female	24	15	7	2
**Race/ethnicity**				
Non-Hispanic White	27	15	8	4
Non-Hispanic Other/Unknown	2	0	2	0
**Education level**				
College graduate/some post-college	9	9	0	0
Master’s degree	8	6	0	2
MD/DO	12	0	10	2
**Tobacco use status**				
Never tobacco user	25	13	9	3
Former tobacco user	3	2	0	1
Unknown (did not wish to answer)	1	0	1	0
**E-cigarette use status**				
Never e-cigarette user	29	15	10	4
**No. of work hours/week,** ***M*** **(SD)**	35.0 (10.9)	29.7 (7.3)	40.3 (13.5)	41.3 (5.5)
**No. of patients cared for/week,** ***M*** **(SD)**	51.0 (34.0)	55.1 (38.2)	57.0 (28.7)	20.3 (7.8)
**No. of years in current position,** ***M*** **(SD)**	8.1 (8.5)	8.6 (9.6)	9.2 (8.0)	3.3 (2.8)
**No. of years at the hospital in any position,** ***M*** **(SD)**	14.1 (8.4)	13.1 (8.5)	13.7 (6.9)	19.0 (11.7)
**Past year training on tobacco counseling**				
No	26	14	8	4
Yes	1	0	1	0
Don’t know	2	1	1	0

^a^*n* unless noted otherwise

Tables 3, 4, and 5 present TDF domains, sub-themes, and exemplar quotes that emerged regarding factors that influence clinical behaviors and providing the “5 A’s” of tobacco counseling during PED/UC visits. The *optimism* and *goals* domains had the fewest sub-themes with only one per each domain, followed by the *reinforcement* and *emotion* domains with two sub-themes. The domains with three sub-themes were *skills*; *social/professional role and identity*; *beliefs about consequences*; *intentions*; *memory, attention, and decision processes*; *social influences*; and *behavioral regulation*. The *knowledge*, *beliefs about capabilities*, and *environmental context and resources* domains each had the most (four) sub-themes.

**Table 3:** Factors influencing clinical behaviors related to tobacco counseling among PED/UC professionals overall and by professional group: knowledge, beliefs about capabilities, and environmental context and resources TDF domains

TDF domain	Sub-theme	Professional group			Sample responses
		Nurse	Physician	Administrator	
Knowledge	Lack of knowledge on tobacco counseling	**✓**	**✓**	**✓**	“I could benefit from education… if I identify someone as a smoker now, I don’t have resources to hand them and I don’t know where to point them.” *— Nurse*
	Lack of procedural knowledge on implementing the “5 A’s” during visit	**✓**	**✓**	**✓**	“One of the things I wasn’t sure about was whether there was an automatic triage question that our registration folks or the triage nurses would ask about secondhand smoke…” — *Physician*
	Not enough information available on cessation resources/referrals	**✓**	**✓**	**✓**	“I think it’s just knowing what’s available. I don’t know what’s out there for smokers.” — *Administrator*
	Not enough information available on thirdhand smoke	**✓**	**✓**	**-**	“I’ll say even if you go outside to smoke, you’ve got to make sure that you wash all of that off of you before you touch your child. I have read a lot about thirdhand [smoke] and that can contribute to their infection… But I don’t feel like we have enough evidence… in our ammunition to basically back ourselves up. I mean there is evidence, but it’s not given to us as nurses to learn to portray in our daily [practice]. It should be, but it’s not one of the modules.” — *Nurse*
Beliefs about capabilities	Not comfortable discussing tobacco counseling with parents	**✓**	**✓**	**✓**	“I think we’re all in healthcare for a reason to make each patient have a better outcome, but if it’s not something that you’re comfortable with reviewing then I feel like it’s just not easy to talk about unless you know exactly… what you can give to them for education.” *— Administrator*
	Easier to discuss with parents who are receptive to tobacco counseling	**✓**	**✓**	**✓**	“From the beginning, if you’re having parents who aren’t interested [in] getting that information, [or] that’s willing to learn that material, I feel like you can attach it to the resource at the end about secondhand smoke and things like that.” — *Administrator*
	Easier to ask and advise when patients have a TSE-related complaint	**✓**	**✓**	**✓**	“I think if it’s pertinent to what’s going on with your child it’s a whole lot better, but if you’re there for stitches me sitting there lecturing you on how you need to quit smoking probably is not the best time. I think if you can tie it into what’s going on, it is probably going to be better received than just me lecturing you.” — *Nurse*
	Would be more likely to discuss parental tobacco use if there were available guidelines and resources/referrals	**✓**	**✓**	**✓**	“Having a trigger come up if they’ve already been asked or screened in triage to discuss it or that the family is open to discussing it. I [don’t] know how to prescribe [nicotine] replacement therapy… Having some sort of work-aid would be helpful where they were asked in triage if they’d be interested and they said yes. Then they would receive counseling and they said that they would be interested in nicotine replacement therapy and all they need is [for] me to prescribe it, or if I had a work-aid that quickly gave me the highest contraindications starting that sort of therapy, I think I would be comfortable writing a prescription for a parent… “ *— Physician*
Environmental context and resources	Lack of time for tobacco counseling	**✓**	**✓**	**✓**	“What makes it challenging in the ED environment is limited time. I mean a lot of the patients I see are with residents… I have the smallest touch point on a patient compared to anyone else; the nurses, the resident. There are some patients that my touch point is a matter of minutes or shorter.” — *Physician*
	Need training and aids to facilitate discussion of sensitive topic with parents	**✓**	**✓**	**✓**	“I would love more training and information on ways to approach families because a lot of times in the ED and UC setting, we don’t have a ton of extra time to do a lot of education with the family. I definitely try to mention [it] during my discharge information if they said that they have been exposed [and] if there is someone that smokes in the house... just advising them that not smoking is very important or definitely being outside and changing your clothes because the exposure to smoking is detrimental to the child. But usually it’s just kind of a quick sentence or two though. So, I would love if we had a handout, resource, dot phrase, or something that we could add into their discharge instructions that we could give them to assist them. Or different ways that I might be more comfortable to address that.” — *Nurse*
	Need cessation resources and referral information to give to parents	**✓**	**✓**	**✓**	“Just handouts and having something where we have quick places that we can go that have resources. Maybe adding it into something where we could flip to that page and say we have these resources, or attachments to the discharge instructions that have links and phone numbers for the families or websites that they could go to would be helpful.” — *Nurse*
	Reason of visit potentially related to child TSE provides a context to offer tobacco counseling	**✓**	**✓**	**✓**	“Since I’m in an ED where we’re looking at mostly a focused encounter and a little general prevention, I almost always ask for identification of smokers [for] most kids with fever, but certainly with respiratory illness… I might do it for some other kids… I don’t do it for every patient. How I do it is generally after I do history of present illness and what’s going on [and] what’s making this worse or what could make it better. The question about what could make this worse is often around other irritants in your environment, other smokers in your home. I don’t usually just let people say, I don’t smoke except outside. I’ll say to them, I think it doesn’t really matter. There is lots of evidence that even things on your hands when you smoke affect your kid, and being outside is not free from having a longer more persistent illness with smoking. So that’s pretty standard practice… I may ask them if I’m getting ready to do their sedation, do you have any asthma or respiratory illness? It may come up then, but it’s not going to come up as part of the reason that they’re in the ED. It’s more about [if they may] have a possible side effect from the treatment because they have more [risk due to] smoke exposure.” *— Administrator*

**Table 4:** Factors influencing clinical behaviors related to tobacco counseling among PED/UC professionals overall and by professional group: skills, social/professional role and identity, and optimism TDF domains

TDF domain	Sub-theme	Professional group			Sample responses
		Nurse	Physician	Administrator	
Skills	Difficulty initiating a discussion with parents and keeping their attention	**✓**	**✓**	**✓**	“What I would need... just a listening ear and their complete attention. That they turn their phone off and don’t look at it for two to three minutes… a visual thing or a stop thing… to break them away from what they’re spending their attention on.” *— Nurse*
	Ability to ask and advise parents of children who present with a respiratory-related complaint or smell like smoke	**✓**	**✓**	**✓**	“So, I wouldn’t say I’m very consistent, but certainly on the kids that are either repeat asthmatics that are there frequently or kids that are sicker. The parents will often say, well I do smoke but I smoke outside, and so I always will kind of go through and talk about well when you come inside [do] you… wash your hands? Do you take off whatever clothes you were wearing outside? A lot of people are very surprised by the amount of smoke that can come in on clothing...” — *Physician*
	Difficulty providing counseling (assess, assist, arrange) due to lack of training and resources	**✓**	**✓**	**✓**	“We don’t really get trained on any ways to help them besides telling them not to do it, so maybe being able to get them an actual resource on quitting or different… outlets that they can look into if they’re really interested in quitting. I don’t feel like we really have any of those resources set up as far as I know right now.” *— Nurse*
Social/professional role and identity	All professional groups should be involved, but need training	**✓**	**✓**	**✓**	“Yes, the physicians and the nurses should have a part in this as long as we’ve been educated and are given the right communication and right resources to offer assistance.” — *Administrator*
	Professional boundaries of stabilizing acute care first	**✓**	**✓**	**✓**	“Unfortunately, my primary goal at that immediate time is to treat the pediatric patients, and... if I have a lot going on, I might just jump in and jump out without being able to spend the amount of time to appropriately counsel parents about those issues.” *— Physician*
	Do not want to pass judgement on parents and make them defensive	**✓**	**✓**	**✓**	“I think it’s really hard when the parents are defensive and they don’t really want to hear it from me, and you kind of feel like you’re overstepping your boundaries a little bit.” *— Nurse*
Optimism	Optimistic that their respective professional group should be involved	**✓**	**✓**	**✓**	“I think anybody who is participating in patient care should be advocating for patients [and] should be involved. I think it can be addressed at multiple levels in their care from triage to disposition or discharge instructions. And so really any nurses, patient care assistants, medics, physicians, [and] students along the way.” — *Physician*

**Table 5:** Factors influencing clinical behaviors related to tobacco counseling among PED/UC professionals overall and by professional group: emotion, beliefs about consequences, and goals TDF domains

TDF domain	Sub-theme	Professional group			Sample responses
		Nurse	Physician	Administrator	
Emotion	Sensitive topic to discuss with parents	**✓**	**✓**	**✓**	“A lot of times in the ER, parents are having the worst day and they already have a lot going on. Most of them want the best for their children. So, it doesn’t always feel like the right time to bring up telling them to stop dealing with the habit they have that helps them deal with their stress.” — *Nurse*
	Stressed to complete tasks during visit	**✓**	**✓**	**✓**	“Clinic flow in the ER is nuts. It’s paramount, right? You often have patients waiting for six hours to be seen and be coming for a completely different problem. Spending too much time on something that is not specifically focused on their chief complaint is a no-no. Time is a precious resource in the ER, so we have to be very efficient in order [for it] to be widely implemented.” *— Administrator*
Beliefs about consequences	When discussing tobacco use and TSE, parents might feel defensive	**✓**	**✓**	**✓**	“We don’t want people to be defensive. We are constantly drilled about patient satisfaction and so it’s hard to have those conversations when it feels confrontational, and then you don’t want them to be upset with you or damage the relationship that you have with a parent. I only address it if it seems like they would be willing to listen or if it seems to be maybe [a] part of the cause of the problem… but other than that I typically don’t say a lot.” *— Nurse*
	Think that not addressing TSE will result in decreased child health and increased repeat visits or hospitalizations	**✓**	**✓**	**✓**	“If you don’t intervene and get buy in from them and get some commitment to change, you may lose the opportunity to improve their overall clinical picture... potentially lead[ing] to decreased need for accessing the health care system [and] decreased exacerbations for asthma if that’s what they’re there for. I see it as missed opportunities to improve their overall healthcare picture.” *— Physician*
	Think that not addressing TSE will reduce the likelihood for parents to quit smoking and in turn children remain exposed and may become smokers themselves	**✓**	**✓**	**-**	“If you’re exposed to stuff in your household, you think it’s okay. Then you grow up doing it as well, so the pattern just kind of continues.” — *Nurse*
Goals	Discussing tobacco counseling with parents using a standardized approach is important	**✓**	**✓**	**✓**	“I definitely think there needs to be some sort of standardized information that you would go through so that it is being presented the same across all boards.” *— Physician*

### Knowledge domain

Specific to the *knowledge* domain, healthcare professionals observed the barrier that they had a lack of knowledge in both (1) tobacco counseling and (2) how to implement counseling into the PED/UC visits (see Table 3). They also observed that they need more information on (3) smoking cessation resources and referral options and (4) thirdhand smoke education to provide to families. Specific to thirdhand smoke, all three professional groups noted that when they perform the “advise” step, they explain the importance of taking proper precautions after smoking (e.g., changing clothes, washing hands) to protect children from thirdhand smoke found on smokers’ clothes and skin. PED/UC professionals also noted that parents, especially those who try to protect their children from secondhand smoke by smoking outside of the home and not around the child, are typically surprised by this information. Professional group differences were found in the *knowledge* domain sub-theme regarding availability of information on thirdhand smoke. Nurses and physicians identified the barrier of lacking educational materials to provide to parents about thirdhand smoke to reinforce the information they presented while administrators did not.

### Beliefs about capabilities and environmental context and resources domains

The *beliefs about capabilities* domain also had four specific sub-themes (see Table 3). Overall, PED/UC professionals reported the (1) barrier that they were uncomfortable with discussing tobacco counseling with parents, (2) enabler that it is easier to have discussions about parental tobacco use and child TSE when the parents are open and receptive to counseling, (3) enabler that it is easier to discuss tobacco use and TSE when the child has a TSE-related chief complaint (e.g., cough) or illness (e.g., asthma), and (4) enabler that they would be more confident and likely to discuss parental tobacco use if there were available guidelines, smoking cessation resources, and referral options to provide to the parents during the visit.

The *environmental context and resources* domain revealed similar but distinct themes. PED/UC professionals suggested they need the following enablers: (1) tobacco cessation resources and referral information to give to parents, (2) training and aids to facilitate discussion of the sensitive topic of tobacco use with parents, and (3) the child’s reason of visit to be potentially related to TSE to provide an opportunity and context to offer tobacco counseling to parents during the visit (see Table 3). Additionally, (4) the barrier of lack of time for prevention in the PED/UC environment was noted as the biggest obstacle to providing tobacco counseling.

### Skills, social/professional role and identity, and optimism domains

The next set of domains, presented in Table 4, that emerged during interviews were *skills*, *social/professional role and identity*, and *optimism.* Concerning the *skills* domain, (1) all PED/UC professional groups reported the barriers of difficulty initiating a discussion about tobacco use with parents, and after identifying parental smokers, difficulty keeping their attention. Healthcare professionals also observed the enabler that they were (2) more skilled in asking about tobacco use and advising parental smokers of children who presented with a TSE-related complaint or smelled like smoke, but observed the barrier that they were (3) less skilled in counseling and assessing parents’ willingness to quit smoking, and assisting/arranging them with cessation support.

For the *social/professional role and identity* domain, (1) PED/UC professionals identified the barrier that discussing tobacco use behavior may come across as passing judgment on parents, thus, making parents defensive. Although all professional groups identified that (2) training all professional groups would enable implementation, (3) they also identified professional boundaries as a barrier since their primary role is to provide acute care to PED/UC patients. The *optimism* domain belief shared by all professional groups was that their respective group should be involved in tobacco counseling efforts.

### Emotion, beliefs about consequences, and goals domains

While only two sub-themes emerged for the *emotion* domain, it is important to note that most PED/UC professionals, across groups, shared two barriers to implementation: (1) tobacco use is a sensitive topic to discuss with parents and (2) they are already stressed to complete tasks related to stabilizing acute care of their patients during visits (Table 5). Many professionals used the words “defensive,” “offended,” “attacked,” and “threatening” while describing how they perceived parents’ emotions while discussing their tobacco use behavior during past PED/UC visits. Most PED/UC professionals in all three groups shared a *belief about consequences* that a barrier to implementation is that parents may be defensive. To avoid making parents defensive and non-receptive, PED/UC professionals in all three groups described using a universal, standardized approach as an enabler of their *goals* to discuss tobacco counseling with parents (see Table 5). Another *beliefs about consequences* sub-theme discussed by all groups was that not addressing parental tobacco use and child TSE will decrease the overall health of PED/UC patients. Only nurses and physicians discussed the perceived consequence that not addressing tobacco use and TSE increases the potential for patients to become smokers in the future.

### Intentions; memory, attention, and decision processes; social influences; behavioral regulation; and reinforcement domains

The remaining domains that emerged during interviews presented in Table 6 were *intentions*; *memory, attention, and decision processes*; *social influences*; and *behavioral regulation*. Specifically, PED/UC professionals stated that their *intentions* to screen for parental tobacco use and child TSE and advise parental smokers to quit smoking are higher when (1) the patient presents with a TSE-related complaint and illness and (2) the patient’s room smells like smoke. However, (3) their *intentions* to screen and counsel are lower when they have competing time demands of stabilizing acute care and fast patient turnover time. Further, all PED/UC professional groups reported the *memory, attention, and decision processes* domain sub-theme that (1) the topic of tobacco counseling was not thought of unless their patient presents with a TSE-related complaint and illness. Only nurses and physicians identified two of the *memory, attention, and decision processes* domain sub-themes as barriers: (2) the topic of tobacco counseling was not thought of unless the patients’ room smells like smoke and (3) there are no reminders to provide tobacco counseling during visits.

**Table 6:** Factors influencing clinical behaviors related to tobacco counseling among PED/UC professionals overall and by professional group: intentions; memory, attention, and decision processes; social influences; behavioral regulation; and reinforcement TDF domains

TDF domain	Sub-theme	Professional group			Sample responses
		Nurse	Physician	Administrator	
Intentions	Intentions to Ask and Advise are higher when patients have TSE-related symptoms and illnesses	**✓**	**✓**	**✓**	“I generally focus on patients who come in for respiratory diseases such as asthma, but it’s not a routine question that I ask.” — *Administrator*
	Intentions to Ask and Advise are higher when patients’ clothing or room smell like smoke	**✓**	**✓**	**✓**	“I usually tend to talk about it more if they actually go out for smoke break or if they smell like smoke in the ED.” — *Physician*
	Intentions to provide counseling are lower when there are competing time demands of stabilizing acute care with fast patient turnover time	**✓**	**✓**	**✓**	“The biggest thing is time. It’s not like it’s inpatient where it’s like, oh I can’t do it now… they’re going to be here all night [so] I’ll do it after rounds this evening. We don’t have that luxury [and] sometimes we really need the patient to be discharged so we can have the room.” — *Nurse*
Memory, attention, and decision processes	Topic is not thought of unless patient has TSE-related complaint or illness	**✓**	**✓**	**✓**	“It is more forefront in my mind when the disease process that I am seeing the child for is related to smoke. Not because I don’t want to do it the rest of time, but because it doesn’t occur to me to do it the rest of the time as clearly.” — *Physician*
	Topic is not thought of unless room smells like smoke	**✓**	**✓**	**-**	“I don’t necessarily screen everybody. I would say it would be more if you smell smoke in the room... Something like that usually triggers me to ask more about social history versus somebody there for an injury.” — *Nurse*
	Not reminded to screen for and counsel parental smokers	**✓**	**✓**	**-**	“I think the more we talk about things, the more they come to mind in a clinic visit. Sort of having something that reminds me to talk about it in clinic or talk about it in the UC. Also, I think [electronic medical record program] reminders.” — *Physician*
Social influences	Parents do not want tobacco counseling	**✓**	**✓**	**✓**	“People get very defensive about it. They lie straight to your face… [I’ve] had babies in distress and I can smell the cigarette smoke on the caregiver that brings them in and [say], you really can’t smoke in the car with them in the car, and they’ll say they didn’t.” *— Nurse*
	Do not know what motivates parents’ smoking behavior	**✓**	**✓**	**✓**	“I think you have to be delicate about it because it’s a lot of people’s vice. It’s very hard to give up, and so I think you have to kind of be mindful of that. You’re asking a lot for someone to give up smoking when most of the time people are doing it because that’s what relieves them.” *— Nurse*
	Difficult to build rapport with families in the acute care setting	**✓**	**✓**	**✓**	“I have to be willing to sit down and talk to them. I can’t stand and lecture with my hands on my hips at the door. I have all the things that I think are more about rapport development and about them seeing you as a person and not as a preacher telling them to do something. I think that’s important. I think from an ED [perspective], it’s important how stressed they are by this specific encounter, [and] what they’re worried about that day. So that is going to often either increase the distance for them or maybe help depending on how open they are to things.” *— Administrator*
Behavioral regulation	Require screening for parental tobacco use	**✓**	**✓**	**✓**	“I think things that would encourage people would be a standardized approach or some sort of checklist that you went through with your discharge instructions [with] a little box or something that would pop up and remind you in [the electronic medical record program] that it’s been noted that the patient is exposed to secondhand smoke to encourage counseling.” *— Physician*
	Receive training with discussion aids	**✓**	**✓**	**✓**	“I think definitely providing a concise resource for the providers to go to [for] handouts, phone numbers for resources, and website links [available in the electronic medical record program]. Maybe even an online course or a 30-minute or 1-hour course about how we can be effective… even ways to maybe bring up the topic… like one liners that you could open up with to get the conversation going with parents.” *— Nurse*
	Having electronic information available to provide to parents	**✓**	**✓**	**✓**	“Nowadays, people don’t want anything on paper and we should decrease paper waste… through some online resources, things like that. I think having tons of handouts is probably not ideal because I think I see more people throw them away… a video to watch would probably be a good thing. Something short and sweet that talks about the harmfulness of it.” *— Nurse*
Reinforcement	Implementing screening questions into the routine clinical flow	**✓**	**✓**	**✓**	“I think if that was built into the triage or screening questions, or if there was a practice alert that popped up when you opened the chart of a patient whose parent identified as a smoker who is interested in getting information, I think that would help.” *— Physician*
	Receiving feedback on the clinical benefit of tobacco counseling	**✓**	**✓**	**✓**	“We know the benefits of not smoking and not being around secondhand smoke… I think if we had something that we could present to them, a tool to help them get passed that, that would be more than enough of an incentive because we know that we’re making an impact on that child’s health.” *— Nurse*

All PED/UC professional groups discussed *social influences* and shared their reluctance to ask about parental tobacco use. Specifically, PED/UC professionals reported the following barriers that they (1) believed parental smokers lack interest in receiving tobacco counseling, (2) do not know what motivates parents to smoke tobacco, and (3) find it difficult to build rapport with parents during their child’s visit. Overall, the professional groups perceived the following would provide them with *behavioral regulation*: (1) requiring screening for parental tobacco use, (2) receiving tobacco use counseling training and discussion aids, and (3) having electronic information to give to parents. The three PED/UC professional groups also discussed that (1) implementing tobacco and TSE screening questions into the routine clinical flow and (2) receiving feedback on the PED/UC patients’ clinical benefit of providing tobacco use counseling to their parents would *reinforce* the importance of providing counseling to parents who are not their patients.

## Discussion

In preparation for future intervention development, the present study used the TDF and identified PED/UC professionals’ current clinical behaviors related to parental tobacco use and child TSE counseling, influences on this behavior, and perceived roles and responsibilities. All TDF domains emerged during the interviews with nurses, physicians, and administrators, with some variation among professional groups where nurses and physicians shared sub-themes, but administrators did not. Key barriers and enablers were identified across professional groups as outlined below.

The major barriers reported by nurses, physicians, and administrators were lack of knowledge, resources, and training on evidence-based tobacco counseling. These barriers emerged in the *knowledge*; *skills*; *social/professional role and identity*; *beliefs about capabilities*; *reinforcement*; *memory, attention, and decision processes*; *environmental context and resources*; *social influences*; and *behavioral regulation* domains. Our findings confirm past PED/UC research that reported limited general knowledge about tobacco counseling and available resources [36]. Overall, PED/UC professionals’ adherence to the Clinical Practice Guideline of *Treating Tobacco Use and Dependence* [17] were mixed, and those who performed tobacco counseling usually only performed the “ask” and “advise” steps. This aligns with prior research that indicates ED professionals often “ask” and “advise,” but infrequently proceed to the next three steps [22, 37–39]. Further, PED/UC professionals noted that they are not skilled beyond asking and advising due to the barriers of lack of training and resources. This aligns with the US Surgeon General’s Report on Smoking Cessation [18] that screening for tobacco use is completed during two-in-three clinical visits compared to providing counseling or education to adult tobacco users, which is done about every one-in-five visits.

Encouragingly, all three PED/UC professional groups revealed they would feel more capable and have higher self-efficacy to perform tobacco counseling, especially the “assess” and “assist/arrange” steps, if there were available guidelines, resources, and referral options for parents and families. There are several available online resources and training tools for healthcare providers (e.g., Tobacco Treatment Specialist certification training [40]) and administrators (e.g., Best Practices for Comprehensive Tobacco Control Programs [41]) to facilitate treatment of tobacco use in the clinical settings [42]. One recommended component of PED/UC professional training is motivational interviewing [17], a collaborative, person-centered counseling technique that can be used to assist smokers in exploring and resolving ambivalence about quitting smoking [43]. A systematic review and meta-analysis of RCTs that evaluated the efficacy of ED-initiated tobacco control found that motivational interviewing and booster phone calls increased tobacco abstinence at 12-month follow-up [21]. Thus, training in motivational interviewing and evidence-based resources and referrals may help to alleviate PED/UC professionals’ concerns about engaging parents in meaningful conversations about their tobacco use. All three professional groups discussed the preference for electronic information and resources on quitting (e.g., cell phone texting), rather than paper-based information and resources (e.g., written self-help packet) to provide to parents and families.

Our study also revealed a *knowledge* domain sub-theme that materials and information on thirdhand smoke exposure are not available to give to patients’ families. A priority for programmatic TSE research is to distinguish thirdhand smoke exposure-specific health risks from secondhand smoke exposure health risks [7]. Current research aims to address the existing knowledge gap on the clinical effects of exclusive thirdhand smoke exposure and pollution among PED/UC patients [44]. Emerging evidence-based research and resources on thirdhand smoke exposure could be tailored to the PED/UC setting. These include freely available educational materials at thirdhandsmoke.org (e.g., webinars [45]). Research on the Clinical Effort Against Secondhand Smoke Exposure (CEASE) intervention to address parental tobacco use during primary care visits shows that sensitizing parents to risks of thirdhand smoke exposure during their children’s primary care visits may positively affect their child’s health [46]. Additionally, parents who believe thirdhand smoke exposure harms their children’s health were more likely to have strict and voluntary home and/or car smoking bans and make at least one quit attempt 12-months later. However, much less is known about offering this type of intervention in the PED/UC setting. The nurse and physician groups in this study indicated that although they verbally share potential health harms of thirdhand smoke exposure with parents (e.g., research showing that PED/UC patients have nicotine on their hands even when no one is smoking around them [47, 48]), they do not have enough evidence-based information on thirdhand smoke to provide to families. Therefore, nurses and physicians expressed a need for materials to provide to families to reinforce what they discussed with them about thirdhand smoke exposure. Further research is needed to establish and test the use of evidence-based materials and messaging on the clinical risks of thirdhand smoke exposure in the PED/UC setting.

Another barrier perceived by all three professional groups is the lack of a standardized protocol for implementing tobacco counseling during the PED/UC visit, which emerged in the *reinforcement*; *intentions*; *goals*; *memory, attention, and decision processes*; and *behavioral regulation* TDF domains. PED/UC professionals in all groups mentioned that requiring screening for parental tobacco use and child TSE would objectively change their behavior to initiate tobacco counseling. A barrier cited by all PED/UC professional groups is that they were not reminded to screen and counsel during the visit. To overcome this barrier, the three PED/UC professional groups suggested the need to implement routine parental tobacco use and child TSE screening questions into the PED/UC flow. Prior qualitative work with ED nurses and physicians suggested the need for acute healthcare systems to implement standardized tobacco counseling practice policies, including incorporating tobacco control interventions into the clinical flow and clarifying professionals’ roles and responsibilities in offering these interventions [49]. All professional groups in this study noted that their immediate role is to stabilize acutely ill patients, and this is a barrier to conducting tobacco screening and counseling during every visit. However, all professional groups were enthusiastic about being involved in tobacco counseling and perceived that their respective groups should be involved and trained in offering tobacco counseling.

Another major barrier cited by PED/UC professionals was that they did not want to seem judgmental towards parental smokers as this may make parents defensive. This barrier emerged during the *skills*, *social/professional role and identity*, *beliefs about consequences*, *intentions*, *goals*, *environmental context and resources*, *social influences*, and *emotion* domains. Thus, having a standardized system in place could assist in determining when to and who should routinely screen for child TSE and/or offer tobacco counseling to tobacco users. Similar to other research [49], smokers’ resistance was frequently cited as a perceived barrier to providing tobacco counseling. Another shared perception of all three professional groups was difficulty initiating a discussion about tobacco use with parents and keeping their attention. For example, this study had reports of parents being on their cell phones during their child’s entire visit. Thus, PED/UC professionals perceived their attention span and body language as non-verbal cues of lack of interest in receiving tobacco counseling. This parallels qualitative research in the adult ED setting that reported assessing non-verbal cues (e.g., rolling eyes when topic is brought up) to gauge patients’ receptiveness to tobacco counseling [49]. The current study’s results underscore the need for a standardized approach to delivering tobacco counseling interventions in the PED/UC setting. This approach could include asking all parents about their child’s TSE status with the triage questions, determining their receptivity and motivation, and tailoring interventions based on their response.

PED/UC professionals identified that leveraging a potential TSE-related complaint (e.g., cough) as a context to provide tobacco counseling to parents would further enable their screening and counseling behaviors. PED/UC professionals frequently stated that they have an easier time asking and advising receptive parents about their child’s TSE, especially those who present with a TSE-related complaint (e.g., cough, asthma), compared with resistant or unresponsive parents. All three professional groups felt skilled in and had increased *intentions* to ask and advise parents of patients who presented with a TSE-related complaint and/or if the room or patient smelled like smoke. Acute healthcare studies have also shown that providers typically ask about tobacco use when patients present for health conditions (e.g., respiratory illnesses) related to smoking [39], and TSE [36]. The current study’s findings expand on these studies by also noting the smell of thirdhand smoke residue deposited on children and their parents’ clothes and skin, as an important enabler of their intentions to provide tobacco counseling. While children presenting with a TSE-related complaint or illness and the room smelling like smoke are reminders to screen for child TSE, universal screening for child TSE is recommended during each pediatric visit [50]. One potential strategy is the use of clinical decision support system (CDSS) tools that can be seamlessly incorporated into the PED/UC flow and can provide rates of TSE screening and tobacco use counseling via electronic medical record queries [51]. A CDSS could facilitate universal screening and counseling based on the “5 A’s” steps, which may mitigate the barrier of parents being defensive or feeling “singled out.” Therefore, future interventions should test ways to screen for child TSE during every visit, and assess CDSS use rates.

Two additional barriers to implementing interventions cited by all professional groups were lack of (1) time during the visit and (2) available PED/UC-based resources. These barriers emerged in the *beliefs about capabilities*; *reinforcement*; *intentions*; *memory, attention, and decision processes*; *environmental context and resources*; and *emotion* domains. *Intentions* to provide counseling were lower when PED/UC professionals had competing acute care-related time demands coupled with a fast patient turnover time. Also, the stress of completing acute care-related tasks during the visit due to time constraints was also identified. Lack of time and resources have been widely cited among ED/PED-based general preventive intervention research (e.g., vaccinations) [11] and tobacco control research [36, 38, 52, 53].

Healthcare professionals can make a difference in increasing overall tobacco abstinence rates with minimal, low-intensity counseling interventions of less than three minutes [17]. Evidence indicates that ED-based screening, brief intervention, and referral to treatment (SBIRT) programs can be cost-effective and cost-beneficial for substance use disorder management among at-risk patients [54, 55]. The PED/UC setting has been used to successfully deliver brief cessation counseling to parental smokers using the SBIRT approach; results indicate that these brief counseling sessions resulted in increases in quit attempts and decreases in tobacco use among parents [56]. An RCT conducted at four EDs in Hong Kong found that brief advice of around one minute that included a message about high smoking-related mortality risks, advice to quit, and referring adult patients to quitline services increased biochemically validated quit rates up to 12-months later, compared to the control group that received a tobacco cessation leaflet [57]. Another potential strategy to reduce the barrier of lack of time is to briefly introduce tobacco counseling to parents during the visit and assisting/arranging them with an active e-referral to a tobacco quitline [58]. Additionally, using a team-based approach and including other PED/UC staff (e.g., social worker) into tobacco efforts would expand available PED/UC-based resources. For example, prior research indicates that mental health counselors can be effective in providing brief interventions for substance use disorders [59], and a computerized tobacco program promoted treatment initiation [60]. Therefore, a multi-disciplinary team-based approach should be considered for delivery of future interventions.

All three professional groups expressed that a consequence of not addressing child TSE during the visit is decreased overall health and repeated PED/UC visits or hospitalizations. These concerns are supported by prior PED/UC research which found that when compared with unexposed children, tobacco smoke-exposed children are at increased odds of having higher resource and medication utilization during visits and are more likely to be admitted to the hospital [61]. Additionally, exposed PED/UC patients are at increased risk of having higher PED costs at their initial visit, followed by greater UC visits and hospitalizations 12-months following their initial visit [62]. Among exposed PED/UC patients only, those with higher cotinine levels had increased risk of having PED visits and hospital admissions over 6-months [63]. PED/UC nurses and physicians also expressed the concern that their patients may initiate smoking in the future, which is also supported by evidence [64]. Therefore, another potential strategy to encourage implementation is to include feedback on the clinical benefit of intervening with families during the PED/UC visit. For example, it may be helpful to provide PED/UC professionals with a summary of the number of children with TSE they identified and parental tobacco cessation rates 6-months following the initial visit. Therefore, providing information on the clinical benefits of intervention (e.g., reductions in the number of tobacco smoke-exposed children who had repeat PED/UC visits or hospitalizations 6-months following their initial visit) should be included in future interventions.

### Limitations

The current study’s limitations should be noted. This study was a sample of PED/UC professionals at one large, Midwestern children’s hospital where a future intervention will be developed and implemented. Therefore, professionals’ views may differ from the general PED/UC professional population’s views. Additionally, some PED/UC professionals were familiar with the study team’s tobacco control research, which may have skewed perceptions and their current practices (e.g., sharing the study team’s work on thirdhand smoke exposure). Further, our qualitative synthesis and results showed that there may be connections between TDF domains (e.g., *knowledge* and *beliefs about capabilities*), but the TDF does not allow for such examination of these links since there are not validated measures to assess associations. However, future research should assess compliance with the “5 A’s” as this will elucidate adherence with the recommended TSE screening and counseling practices. Future research should also consider observing PED/UC professionals during the visits.

## Conclusions

This study’s findings support the need to develop and implement an intervention to support PED/UC professionals in their tobacco prevention and control practices. The TDF provided rich, valuable qualitative data to understand current clinical behaviors in following the Clinical Practice Guideline of *Treating Tobacco Use and Dependence* [17] and provided a framework for future intervention development and implementation. Thus, the planned intervention will address the range of barriers through use of the enablers identified during interviews with PED/UC professionals. Sample intervention components include a standardized approach using a CDSS within the electronic medical record delivered during optimal times within a visit, brief counseling that uses motivational interviewing techniques, a team-based approach for intervention delivery, and providing feedback reports to the healthcare team on the benefits of the intervention on child and parental health. Intervention development and implementation plans will address all TDF domains, include tobacco training, and test the most effective methods, resources, intensity, and timing of intervention delivery in the PED/UC setting.

## Data Availability

The dataset generated and/or analyzed during the current study are not publicly available due to the potential risk of identification of participants, but a limited dataset is available from the corresponding author on reasonable request.

## Abbreviations

ED: Emergency department
PED: Pediatric emergency department
UC: Urgent care
TSE: Tobacco smoke exposure
TDF: Theoretical domains framework
IRB: Institutional review board
RCT: Randomized controlled trial
